# Rift Valley Fever Virus Primes Immune Responses in *Aedes aegypti* Cells

**DOI:** 10.3390/pathogens12040563

**Published:** 2023-04-06

**Authors:** Mathilde Laureti, Rui-Xue Lee, Amelia Bennett, Lucas Aladar Wilson, Victoria Elena Sy, Alain Kohl, Isabelle Dietrich

**Affiliations:** 1 The Pirbright Institute, Ash Road, Pirbright GU24 0NF, UK; 2 MRC-University of Glasgow Centre for Virus Research, 464 Bearsden Road, Glasgow G61 1QH, UK; 3 Department of Life Sciences, Faculty of Science, Claverton Down, University of Bath, Bath BA2 7AY, UK; 4 Institute of Medical Sciences, School of Medicine, Medical Sciences and Nutrition, Foresterhill, University of Aberdeen, Aberdeen AB25 2ZD, UK

**Keywords:** mosquito immunity, Rift Valley fever virus, immune priming

## Abstract

The ongoing global emergence of arthropod-borne (arbo) viruses has accelerated research into the interactions of these viruses with the immune systems of their vectors. Only limited information exists on how bunyaviruses, such as Rift Valley fever virus (RVFV), are sensed by mosquito immunity or escape detection. RVFV is a zoonotic phlebovirus (Bunyavirales; *Phenuiviridae*) of veterinary and human public health and economic importance. We have shown that the infection of mosquitoes with RVFV triggers the activation of RNA interference pathways, which moderately restrict viral replication. Here, we aimed to better understand the interactions between RVFV and other vector immune signaling pathways that might influence RVFV replication and transmission. For this, we used the immunocompetent *Aedes aegypti* Aag2 cell line as a model. We found that bacteria-induced immune responses restricted RVFV replication. However, virus infection alone did not alter the gene expression levels of immune effectors. Instead, it resulted in the marked enhancement of immune responses to subsequent bacterial stimulation. The gene expression levels of several mosquito immune pattern recognition receptors were altered by RVFV infection, which may contribute to this immune priming. Our findings imply that there is a complex interplay between RVFV and mosquito immunity that could be targeted in disease prevention strategies.

## 1. Introduction

Arthropod-borne (arbo) viruses replicate and disseminate in their arthropod vector before being transmitted to a susceptible vertebrate host when the infected vector takes a blood meal. In mosquitoes, viral replication initially occurs in midgut epithelial cells before the virus disseminates into the hemocoel and infects other organs, including the trachea, fat body, and, finally, the salivary glands. Mosquito immune responses regulate the outcome of viral infections by modulating the viral load in these tissues and also the extrinsic incubation period and viral pathogenesis in the vector [1]. The insect innate immune response is mediated by four major immune signaling pathways. These include the nuclear factor kappa-light-chain-enhancers of activated B cell (NF-κB)-regulated Toll and immune deficiency (IMD) pathways, the Janus kinase-signal transducer and activator of the transcription (Jak-STAT) pathway, and RNA interference [2,3]. Pathway activation leads to the production of antimicrobial peptides (AMPs) and other effectors, as well as the degradation of virus-derived RNA. 

We previously reported that Rift Valley fever virus (RVFV) infection of *Aedes* spp. and *Culex* spp. mosquitoes induced virus-specific RNA interference (RNAi) and that the silencing of the RNAi machinery in mosquito cells increased the replication of RVFV and that of other bunyaviruses [4,5]. However, the potential role that other innate mosquito immune pathways play in anti-RVFV defense remains to be characterized. 

**Figure 1:**
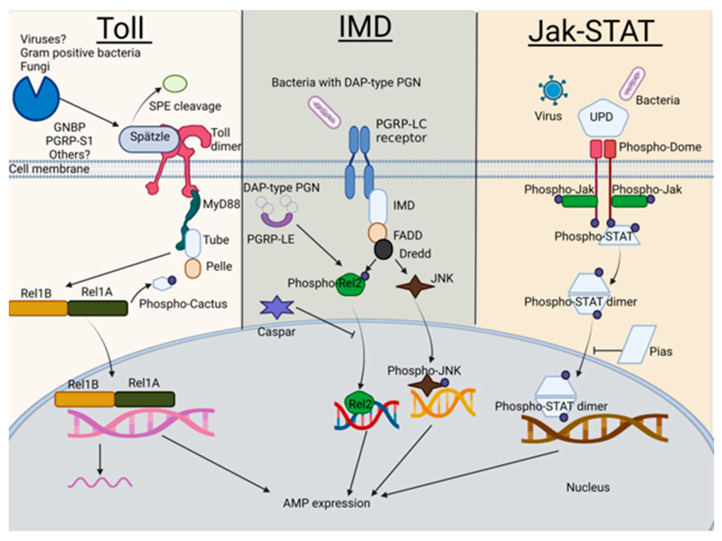
*Ae. aegypti* Toll, IMD, and Jak-STAT pathways. (i) The Toll pathway is activated by the recognition of pathogen-derived ligands by pattern recognition receptors (PRRs), such as Gram-negative binding proteins (GNBPs) and peptidoglycan recognition proteins (PGRPs). Immune challenge, primarily by Gram-positive bacteria and fungi, trigger the proteolytic processing of the cytokine Spätzle, which binds to the Toll receptor and activates signaling [19,20,21]. This leads to the formation of a signaling complex, comprising dimerized Toll and the adaptor proteins Myeloid Differentiation factor 88 (MyD88), Tube, and Pelle [22,23], and causes the phosphorylation and degradation of the negative pathway regulator Cactus [24]. This allows for the translocation of the transcription factor Rel1 into the nucleus, which results in the transcription and translation of antimicrobial peptides (AMPs) and other effectors [25]. (ii) The engagement of the IMD pathway requires the recognition of the diaminopimelic acid-containing peptidoglycan (PGN) present in the cell walls of Gram-negative bacteria by the extracellular peptidoglycan recognition protein LE (PGRP-LE; not shown) in synergy with the transmembrane peptidoglycan recognition protein LC (PGRP-LC) [26,27,28,29,30]. PGRP-LC activates both the c-Jun N-terminal Kinase (JNK) and the Rel2 arms of the IMD pathway [31]. The IMD death domain adaptor protein binds to Fas-associated death domain protein (FADD). Both interact with Dredd, causing the cleavage of phosphorylated Rel2. Activated Rel2 is translocated to the nucleus, leading to the transcription of several *AMP*s. Cytosolic fractions of PGRP-LE also detect intracellular bacteria and activate IMD signaling independently of PGRP-LC [32]. The IMD pathway is negatively regulated by Caspar [33]. JNK signaling promotes the transcription of response genes involved in processes like apoptosis and complement activation. (iii) Upon infection with bacteria or viruses, the binding of an extracellular unpaired peptide ligand (Upd) to the transmembrane receptor Domeless (Dome) results in the activation of the Jak-STAT pathway [34,35]. Dome undergoes a conformational change and dimerization, which causes the self-phosphorylation of associated Janus kinases (Jaks), such as Hopscotch (Hop) [34,36]. Activated Jaks, in turn, phosphorylate the cytoplasmic end of Dome, creating docking sites for STATs [37]. Recruited STATs are phosphorylated and undergo dimerization [38]. STAT dimers are translocated into the nucleus, where the transcription of specific genes is activated (reviewed in [39]). The protein inhibitor of activated STAT (PIAS) negatively regulates Jak-STAT signaling [40]. This figure was created with BioRender.com.

The mosquito Toll, IMD, and Jak-STAT pathways (summarized in Figure 1) have all been shown to respond to arbovirus infections. The Toll pathway was found to be involved in the defense against Gram-positive bacteria [6], fungi [6], and arboviruses [7]. It mediates the mosquito’s anti-dengue virus (DENV; *Flaviviridae*, Flavivirus) defense [8]. The IMD pathway is primarily activated by bacteria, while viral pathogen-associated molecular patterns (PAMPs) and mosquito IMD pattern recognition receptors (PRRs) that sense viruses remain largely uncharacterized. A recent study by Russell et al. (2021) has shown IMD pathway activation in *Ae. aegypti*-derived cells by dsRNA, which is known to arise during viral replication in insect cells or is present in viral RNA genomes in the form of secondary structures. The same study also confirmed IMD activation following the infection of cells with cricket paralysis virus (CrPV; *Dicistroviridae*, Cripavirus) [9], while microbiota-mediated IMD stimulation decreased Sindbis virus (SINV; *Togaviridae*, Alphavirus) loads in *Ae. aegypti* [10]. Further, the silencing of the transcription factors Rel2 (IMD) or STAT (Jak-STAT) in *Anopheles gambiae* increased midgut loads of o’nyong’nyong virus (ONNV; *Togaviridae*, Alphavirus) [11]. The infection of *Ae. aegypti* mosquitoes with a range of flaviviruses led to the upregulation of Jak-STAT response genes [12,13]. Studies have shown that the silencing of Jak-STAT pathway components increases the susceptibility of *Ae. aegypti* to DENV [14]. Further, the pathway mediates the immunity of *An. gambiae* and *Ae. aegypti* to bacteria [15,16], as well as to the protozoan parasites *Plasmodium berghei* and *P. falciparum* [17]. It is currently not known if these immune signaling pathways form part of the mosquito immune response to RVFV. 

RVFV cycles between domestic ruminants and mosquitoes, with the occasional infection of humans mainly through contact with infected animals. The virus is transmitted by many mosquito species, which is unique among arboviruses. The emergence of RVFV outside of the African continent has raised concerns about the virus spreading to Asia, Europe, or the Americas. Outbreaks of RVFV can have severe socioeconomic impacts, including significant trade reductions and economic losses [18]. The available preventative measures for use in livestock are often used in outbreak situations only due to concerns around safety profiles and possible reassortment events with circulating virus strains. The production of transgenic mosquitoes with a reduced ability to transmit RVFV is, thus, of current research interest, as this targets the transmission cycle directly. 

In this study, we aimed to elucidate the role of Toll, IMD, and Jak-STAT signaling in RVFV infection of mosquito cells. We first characterized the ability of our isolate of *Ae. aegypti* Aag2 cells to signal through these pathways using heat-inactivated bacteria as a validated stimulant and confirmed that these cells primarily respond to bacteria via the IMD and Jak-STAT pathways. We then showed that RVFV growth was sensitive to immune signaling in Aag2 cells. In contrast, RVFV infection alone did not alter *AMP* gene expression; it primed the immune responses of cells to bacteria, an effect not observed with the closely related Bunyamwera orthobunyavirus (BUNV; Bunyavirales; *Peribunyaviridae*). We found modest changes in the gene expression of selected pattern recognition receptors upon RVFV infection, which may contribute to the observed immune priming. However, further investigation of the exact mechanism(s) is required. This study shows that there is indeed an interaction between RVFV and the innate immune system of mosquitos that may shape the viral replication and transmission dynamics in the mosquitoes. The results from this study will, thus, inform efforts to prevent mosquito virus transmission to ruminants and humans.

## 2. Materials and Methods

### 2.1. Cells and Viruses 

BHK-21 and BSRT7/5 cells were grown in Glasgow’s minimal essential medium (GMEM) supplemented with 10% fetal calf serum (Gibco, Fisher Scientific, Loughborough, UK), 10% tryptose phosphate broth (Gibco), 100 U/mL penicillin, and 100 µg/mL streptomycin (Gibco) at 37 °C in 5% CO_2_. BHK-21 cells were obtained from R. Elliott (MRC-University of Glasgow Centre for Virus Research, Glasgow, UK). BSR-T7/5 cells were a kind gift from K.-K. Conzelmann (Max von Pettenkofer Institute, Ludwig-Maximilians-University Munich, Munich, Germany). *Aedes aegypti*-derived Aag2 cells (kindly provided by P. Eggleston, Keele University, Keele, UK) were cultured in Leibovitz’s L-15 media (Gibco), supplemented with 10% high-performance fetal bovine serum (Gibco), 10% tryptose phosphate broth (Gibco), 100 U/mL penicillin, and 100 µg/mL streptomycin (Gibco), and maintained at 28 °C. AF05 cells were kindly provided by K. Maringer (The Pirbright Institute, Pirbright, UK) and propagated under the same conditions as Aag2 cells. Lipopolysaccharide (LPS; Merck, Gillingham, UK) was used at a concentration of 10 ng/mL. Actinomycin D (Fisher Scientific) was used at 50 ng/mL. Bafilomycin A1 and torin-1 were used at 0.5 µM. 

RVFV rMP-12 as well as NSm and NSs deletion mutants were generated using a reverse genetics system as previously described [41] and kindly provided by M. Bouloy and M. Flamand (Institut Pasteur Paris, Paris, France). Virus stocks were kindly provided by R. Elliott (MRC-University of Glasgow Centre for Virus Research, Glasgow, UK). Bunyamwera virus (BUNV) was rescued as described previously [42]. The reverse genetics system was a kind gift from X. Shi (MRC-University of Glasgow Centre for Virus Research, Glasgow, UK). Briefly, BSR-T7/5 cells were transfected with 0.5 μg each of pT7riboBUNL(+), pT7riboBUNS(+) and TVT7RBUNM(+) cDNA. Cells were incubated for 5 days at 33 °C until cytopathic effect (CPE) was evident. Further propagation of RVFV and BUNV was performed on BHK-21 cells at 33 °C, and virus stocks were titred by plaque assay on BHK-21 cells. All experiments with infectious RVFV were conducted under biosafety level 3 conditions. Virus infections of Aag2 and AF05 cells were performed in L15 medium using indicated multiplicities of infection (MOI) for 1 h at 28 °C. The media was then changed, and cells were incubated for assay-specific amounts of time at 28 °C. 

### 2.2. Bacterial Immune Stimulation and Virus Infections

(i) To induce immune signaling using heat-inactivated bacteria, Aag2 cells were seeded at a density of 3 × 10^5^ cells per well in 24-well plates and left to adhere overnight. Cultures of *Escherichia coli* (strain JM109; Promega, Chilworth, UK) and *Staphylococcus aureus* (ATCC) were grown in 5 mL LB broth without antibiotics and incubated at 37 °C for 16 h. Serial dilutions of the cultures were prepared, and cell forming units per mL were determined on LB agar plates following an overnight incubation at 37 °C. The remainder of the cultures were centrifuged at 1174× *g* for 20 min at 4 °C. The bacterial pellets were washed twice in phosphate-buffered saline (PBS), resuspended in 500 µL PBS, and heat-inactivated for 10 min at 80 °C. Aag2 cells were stimulated with heat-inactivated *E. coli* and *S. aureus* [multiplicity of infection (MOI) of 300 CFU/cell] for 16 h at 28 °C. PBS was used as control. Cells were washed with PBS and lysed in TRIzol Reagent (Life Technologies, Paisley, UK) according to the manufacturer’s instructions;
(ii) To assess the impact of bacterial immune stimulation on viral replication, Aag2 cells were treated with heat-killed bacteria for 16 h. Cells were then infected with RVFV rMP-12 or rBUNV at MOI 0.1 or left uninfected for 24 h, and cells lysed in TRIzol Reagent;
(iii) To quantify the effect of viral infection on immune gene expression and RNA levels of insect-specific viruses, cells were mock-infected or infected with RVFV rMP-12, RVFV rMP-12:delNSm, RVFV rMP-12:delNSs or rBUNV at MOI 1 for 24 h. Cells were lysed in TRIzol Reagent;
(iv) Lastly, to determine if virus infection alters immune gene expression in response to bacterial stimulation of Aag2 cells, cells were mock-infected or infected with RVFV rMP-12 or rBUNV at MOI 1 for 24 h, followed by bacterial or PBS stimulation for 16 h. Cells were lysed in TRIzol Reagent.

As Aag2 cells are sensitive to treatment with heat-inactivated bacteria, especially the bacterial debris which settles onto the cell monolayer over time, and undergo morphological and potentially further poorly-characterized changes that may impact viral replication independently of activation of immune signaling, bacterial stimulation was also conducted using Aag2-derived AF05 cells [43]. These cells seem to overall be more tolerant to stress. Here, commercial preparations of heat-killed *E. coli* and *S. aureus* were used (Invivogen, Toulouse, France). These induced *AMP* expression in Aag2 cells to similar levels to in-house preparations when used at the same MOI (300 CFU/cell). It should be noted that these preparations also led to debris precipitation onto cells during treatment.

### 2.3. Quantification of Gene Expression

RNA was phenol-chloroform extracted and reverse transcribed using Superscript III enzyme (Life Technologies) and random hexamer primers (Promega) according to manufacturer’s guidelines. Expression levels of *Ae. aegypti* immune genes, RNA levels of insect-specific viruses, RVFV, or BUNV were determined by qRT-PCR using Fast SYBR Green Master Mix (Applied Biosystems, Birchwood, UK) according to manufacturer’s instructions on a Quantstudio 3 qRT-PCR cycler (Applied Biosystems) using the recommended cycling conditions and gene-specific primers (Table 1). *Ae. aegypti ribosomal protein S7* (*RPS7*) was amplified as housekeeping gene (Table 1). Primers were designed using Primer3 software [https://primer3.ut.ee/ (accessed on several occasions between 1 October 2015 and 1 December 2022)] using the following parameters: product size range 50–120 nt; primer Tm 60 °C; self and pair complementary scores of zero; end and pair-end complementary scores of zero; primer hairpin formation score of zero. Where possible, amplicons were designed over exon-intron-exon boundaries. Primer pairs were blasted against the *Ae. aegypti* genome to ensure specificity. Primer pair efficiencies were confirmed to be between 90 and 110%. Melting curves were run to exclude dimer formation. Fold changes in gene expression were determined using the comparative ΔΔCT method, and error bars represent standard error of mean of the triplicate experiments.

### 2.4. Plaque Assays

In addition to qRT-PCR, RVFV rMP-12 growth following bacterial stimulation of AF05 cells was quantified by plaque assay on BHK-21 cells for 72 h using cell culture supernatants and 0.6% Avicel (FMC Corporation, Deeside, UK) as overlay. Plaque assays were fixed with formaldehyde (Thermo Fisher Scientific, Basingstoke, UK) and stained with toluidine blue (Thermo Fisher Scientific). 

### 2.5. Pathway Sensors and Luciferase Assays

*AMP* expression following the treatment of cells with heat-inactivated bacteria was also quantified using pathway reporter constructs as described previously [11,44]. Briefly, Aag2 cells were transfected with 12.5 ng pAct-*Renilla* construct expressing *Renilla* luciferase as transfection control [50] and either 500 ng pJM648 (Toll pathway reporter; firefly luciferase under the control of the *Drosophila Drosomycin* promoter), 25 ng pJL195 (IMD pathway reporter; firefly luciferase under the control of the *Drosophila Attacin* A promoter) [51] or 25 ng p6x2DRAF-Luc (Jak-STAT pathway reporter; firefly luciferase under the control of a multimerized *Drosophila* STAT-responsive element) [52] for 24 h using Lipofectamine 2000 (Life Technologies) according to the manufacturer’s protocol. Immune signaling was then stimulated using heat-inactivated bacteria for 16 h, and luciferase activities were determined using the Dual-Luciferase Assay System (Promega) on a GloMax luminometer (Promega).

### 2.6. Caspase Assays

Aag2 cells were treated with heat-inactivated bacteria or PBS for 16 h. Treatment of cells with 50 ng/mL actinomycin D (Fisher Scientific) in DMSO (or DMSO only) for up to 6 h was used as positive control for the induction of apoptosis. Cells were lysed in passive lysis buffer (Promega), and luciferase activity was measured using the CaspaseGlo-3/7 system (Promega) on a GloMax luminometer (Promega). 

### 2.7. Induction of Autophagy and Immunoblotting

To induce autophagy, Aag2 cells were treated with 0.5 µM bafilomycin A1 and 0.5 µM torin-1 for 24 h using 1% DMSO as control. Cells were lysed in passive lysis buffer, and protein concentrations were quantified using the Pierce BCA Protein Assay Kit (Thermo Fisher Scientific). Absorbances were read on a GloMax luminometer. Proteins were separated by SDS-PAGE and transferred using the Trans-Blot Turbo Transfer System (Bio-Rad, Oxford, UK). *Ae. aegypti* ATG8 was detected using a rabbit anti-GABARAP antibody (ab109364; Abcam, Cambridge, UK), and β-actin was detected with a mouse anti-actin antibody (MABT219/JLA20; Merck), both used at 1:1000. Primary antibodies were detected using goat anti-rabbit IgG H&L (HRP) (ab6721; Abcam) and goat anti-mouse IgG H&L (HRP) (ab6789; Abcam) secondary antibodies, respectively. Bands were visualized with ECL reagent (Bio-Rad) on a Bio-Rad ChemiDoc system and Image Lab software (Bio-Rad). Band intensities were analyzed and compared in ImageJ.

### 2.8. Statistical Analyses

Each experiment was carried out a minimum of three times in triplicate. Statistical significances were analyzed as described in figure legends. Asterisks represent significant results (* *p* < 0.05, ** *p* < 0.01, *** *p* < 0.001, **** *p* < 0.0001). All statistical analyses presented were performed using GraphPad Prism (version 9; GraphPad Software, Inc., La Jolla, CA, USA).

## 3. Results

### 3.1. Immune Stimulation of Different Aag2 Cell Isolates Induces AMP Transcription to Varying Degrees

Different isolates of Aag2 cells vary in the degree to which they can be stimulated with heat-inactivated bacteria [43,46,49,53]. The production of AMPs also depends on the stimulus used and the response genes quantified. In order to be able to investigate immune signaling in response to RVFV infection and to be able to compare our findings to those of published studies on immunity in Aag2 cells, we characterized the immune signaling of our Aag2 cell isolate in response to bacteria using (*Drosophila*-derived) Toll, IMD, and Jak-STAT pathway sensors, as described previously [11,44], or by qRT-PCR. Although we saw a significant induction of the IMD and Jak-STAT pathway sensors in response to heat-inactivated *Escherichia coli* bacteria (*p* = 0.029 and *p* = 0.0005, respectively), there was no significant induction in luciferase expression from the Toll pathway sensor construct (Figure S1). 

**Figure 2:**
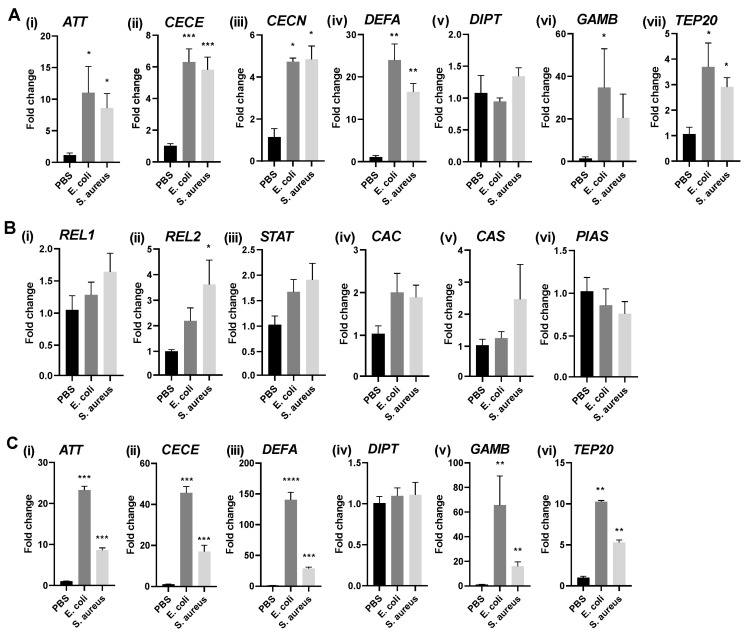
Stimulation of immune signaling in *Ae. aegypti* Aag2 and Aag2-AF05 cells. (**A**) Aag2 cells were stimulated with heat-inactivated *E. coli* or *S. aureus* (prepared in-house) or were left unstimulated (PBS control) for 16 h, and the induction of *AMP* gene expression was quantified by qRT-PCR. (**B**) Aag2 cells were stimulated with heat-inactivated *E. coli* or *S. aureus* (prepared in-house) or were left unstimulated (PBS control) for 16 h, and the induction of expression of transcription factors *REL1* (Toll pathway), *REL2*; IMD pathway), and *STAT* (Jak-STAT pathway), or pathway negative regulators *CAC* (Toll pathway), *CAS* (IMD pathway), and *PIAS* (Jak-STAT pathway), was quantified by qRT-PCR. (**C**) Aag2 cell-derived AF05 cells were stimulated with commercial preparations of heat-killed *E. coli* or *S. aureus* or were left unstimulated (PBS control) for 16 h, and the induction of *AMP* gene expression was quantified by qRT-PCR. The bars represent mean gene expression fold changes in the treated groups versus the controls, with the error bars indicating the standard error of mean (SEM), *n* = 3. Statistical analyses were performed on ΔΔCt values using Welch t-tests, assuming unequal variances. Asterisks represent significant differences between treated groups and respective controls.

The stimulation of Aag2 cells with heat-inactivated *E. coli* and *Staphylococcus aureus* resulted in a significant increase in the expression of different *Ae. aegypti AMP* genes, but not *DIPT* (Figure 2A).

In contrast to the *AMP*s, the expression of the immune pathway transcription factors *REL1* (Toll pathway), *REL2* (IMD pathway), and *STAT* (Jak-STAT pathway), or the pathway negative regulators *CAC* (Toll pathway), *CAS* (IMD pathway), and *PIAS* (Jak-STAT pathway), were not differentially regulated upon bacterial treatment of Aag2 cells (Figure 2B), with the exception of *REL2* upon treatment of the cells with *S. aureus* (Figure 2(Bii)). Further, the levels of persistently infectious, insect-specific viruses that are known to be present in Aag2 cells, cell fusing agent virus (CFAV; *Flaviviridae*, Flavivirus) and phasi charoen-like phasivirus (PCLPV; *Phenuiviridae*, Phasivirus) remained unchanged following immune challenges (Figure S2A). 

In order to investigate how *AMP* expression levels in our Aag2 cells would differ from that in other Aag2 cell isolates, we repeated the stimulations in the Aag2-AF05 cell line [43], which has been derived from Aag2 cells of a different origin to ours. Following treatment with heat-inactivated bacteria, the overall *AMP* gene induction levels were higher in the AF05 cells compared to our Aag2 cells (Figure 2C). The levels of CFAV and PCLPV in the AF05 cells did not change upon bacterial stimulation (Figure S2B). BothAag2 and AF05 cells were negative for Culex Y virus (CYV; *Birnaviridae*; Entomobirnavirus) (not shown), which can infect Aag2 cell cultures [54]. 

### 3.2. Immune Stimulation Reduces RVFV Replication

**Figure 3:**
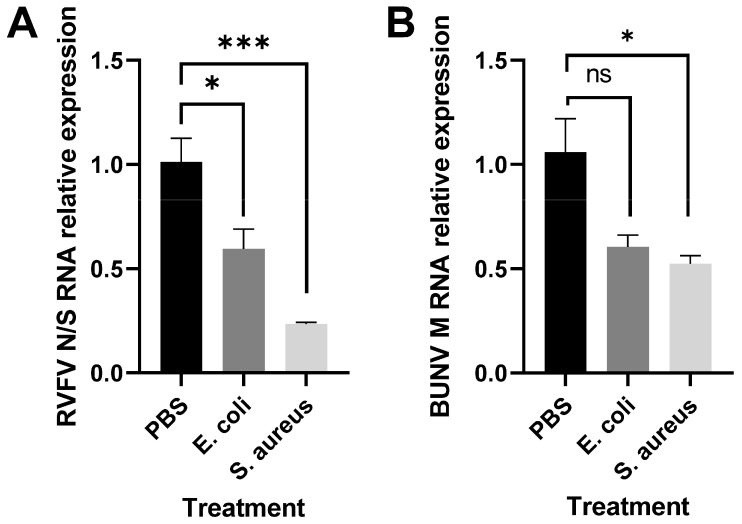
Immune signaling affects RVFV and BUNV replication in Aag2 cells. Aag2 cells were treated with heat-inactivated *E. coli*, heat-inactivated *S. aureus,* or PBS (as control) for 16 h and were infected with (**A**) RVFV rMP-12 or (**B**) rBUNV at MOI 0.1 for 24 h. Viral RNA levels were quantified by qRT-PCR. The bars represent the means of gene expression fold changes in the treated groups versus the controls +/− SEM, *n* = 3. Statistical analyses were performed on ΔΔCt values usingunpaired t-tests.

In order to study the effect of immune stimulation on RVFV replication, Aag2 cells were subjected to heat-inactivated bacteria and subsequently infected with RVFV rMP-12. The prestimulation with bacteria reduced the relative abundance of RVFV RNA (Figure 3A) and also affected the replication of the related orthobunyavirus Bunyamwera (BUNV) (Figure 3B). 

**Figure 4:**
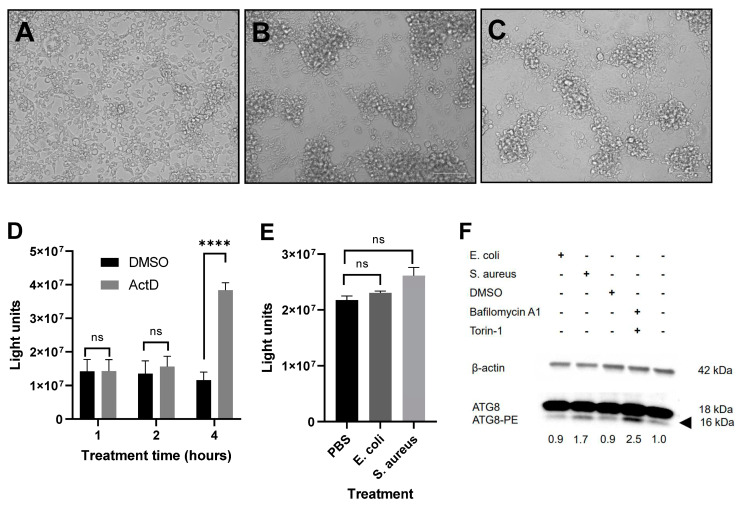
Immune stimulation of Aag2 cells causes morphological changes that are not related to apoptosis or autophagy. Cellular morphology of Aag2 cells following treatment with (**A**) PBS control, (**B**) heat-inactivated *E. coli,* or (**C**) heat-inactivated *S. aureus* for 16 h, recorded on an EVOS FLoid fluorescent microscope (Thermo Fisher Scientific). (**D**) Aag2 cells were stimulated with ActD or DMSO (as control) for indicated periods of time, and the caspase activity in cell lysates was measured by luciferase assay. The bars represent the means +/− SEM, *n* = 3. Statistical analyses were performed using multiple Welch t-tests with Bonferroni-Dunn correction, assuming unequal variances. (**E**) Aag2 cells were stimulated with PBS control or commercial preparations of heat-inactivated *E. coli* or *S. aureus* for 16 h. Caspase activity in cell lysates was measured by luciferase assay. The bars represent the means of triplicate quantification +/− SEM, *n* = 3. Statistical analyses were performed using unpaired t-tests. (**F**) Aag2 cells were either stimulated, as in (**E**), treated with DMSO as the control, or a combination of bafilomycin A1 and torin-1 for 24 h. ATG8 and ATG8-PE expression levels were determined by immunoblotting. β-actin was quantified as the loading control. The black arrowhead indicates ATG8-PE. Numbers below the blot indicate normalized ATG8-PE-to-β-actin band intensity ratios.

We noted that treatment caused Aag2 cells to change their morphology and to aggregate (Figure 4A–C), and this was more pronounced with increasing bacterial load and incubation time (not shown but also reported in [55]), indicating that Aag2 cells were sensitive to treatment and debris settling onto the monolayer. In order to ensure that the reduction in viral replication observed in Figure 3 was not caused by cells entering cell death and, hence, being less able to become infected in the first place or less able to propagate the virus due to reasons other than immune activation, we first established a caspase assay that was able to detect apoptosis in Aag2 cells. For this, we used actinomycin D (ActD) as an apoptosis inducer and a commercially available caspase 3/7 assay that functions through the caspase cleavage of a proluciferin DEVD substrate to release the luciferase substrate aminoluciferin. DEVD has previously been shown to be a suitable substrate for the mosquito effector caspases involved in apoptosis [56,57]. While cells treated with ActD for a minimum of 4 h readily underwent apoptosis (Figure 4D), cells treated with heat-killed bacteria for up to 16 h did not (Figure 4E), despite obvious changes to their morphology (Figure 4B,C).

A second cellular process that is activated by innate immune signaling and cellular stress and that can lead to cell death is macroautophagy. In order to determine if autophagy was induced in response to Aag2 cells’ exposure to heat-killed bacteria, we treated cells with bacteria or a combination of the autophagy inducers bafilomycin A1 and torin-1 (Figure 4F). Expression levels of the lipidated form of the autophagy-related protein ATG8 [ATG8-PE (PE, phosphatidylethanolamine), a marker of autophagosome formation] were quantified by immunoblotting. Bafilomycin A1/torin-1 induced autophagy, as indicated by increased ATG8-PE formation. To a lesser extent, the treatment of cells with *S. aureus* and not *E coli* also induced autophagy. Importantly, however, the activation of autophagy by bafilomycin A1/torin-1 did not result in the cells aggregating (not shown). 

**Figure 5:**
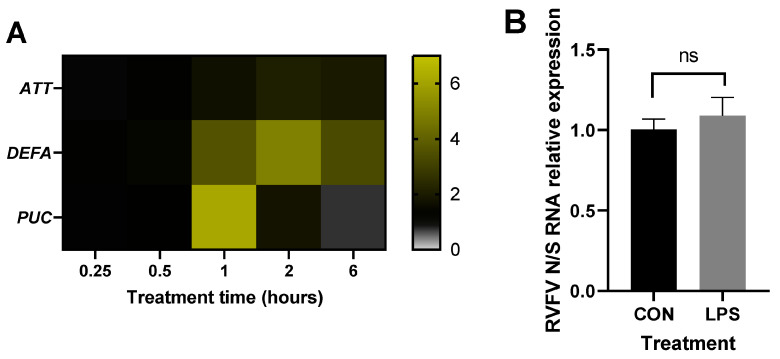
Immune stimulation of Aag2 cells with LPS induces JNK and IMD signaling but does not affect RVFV replication. (**A**) Aag2 cells were treated with LPS or were left untreated for indicated periods of time. The induction of response genes was quantified by qRT-PCR. The heat map represents gene expression fold changes of *AMP*s in treated groups normalized to the controls, *n* = 3. Statistical analyses were performed on ΔΔCt values using multiple Welch t-tests with Bonferroni-Dunn correction, assuming unequal variances. *DEFA* gene expression was significantly upregulated at 1, 2, and 6 h of LPS treatment (*p* = 0.012, *p* = 0.008, and *p* = 0.004, respectively). *PUC* gene expression was significantly upregulated at 1 h of LPS treatment (*p* = 0.0006). (**B**) Aag2 cells were treated with LPS or were left untreated (CON) for 1 h, after which they were infected with RVFV rMP-12 at MOI 0.1 for 24 h. RVFV replication was quantified by qRT-PCR. The bars represent the means of expression fold changes in the treated group versus the control group +/− SEM, *n* = 3. Statistical analyses were performed on ΔΔCt values using an unpaired t-test.

We then sought to induce immune signaling via means that would not affect Aag2 cell morphology to gain more confidence in our finding that immune activation limits RVFV replication (Figure 3A). Lipopolysaccharide (LPS) is a major cell wall component of Gram-negative bacteria. Preparations of LPS often contain traces of peptidoglycan [58] that are capable of activating IMD signaling through JNK and Rel2 arms. The successful activation of JNK and IMD signaling in Aag2 cells by LPS was confirmed by measuring the expression levels of the JNK negative regulator Puckered encoding gene *PUC* and the IMD *AMP*s *ATT* and *DEFA* (Figure 5A). 

Indeed, *PUC* expression as a readout for JNK pathway activation peaked 1 h post-LPS treatment, following which the expression of *ATT* and *DEFA* was induced. Cell morphology was not affected by LPS treatment (not shown). RVFV RNA levels remained unaltered following the pretreatment of the cells with LPS when compared to the nontreated cells (CON; Figure 5B). It should be noted, though, that the *AMP* induction levels using LPS as an immune stimulant were low compared to the heat-killed bacteria (Figure 2A) and were transient. 

**Figure 6:**
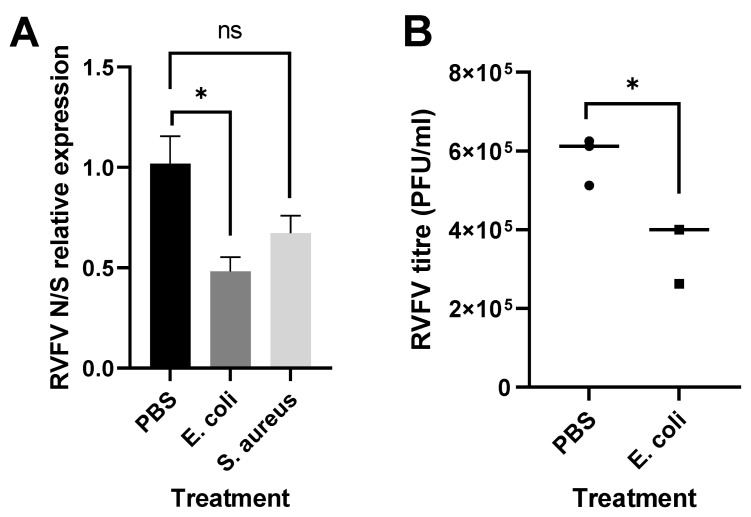
Immune stimulation of Aag2-AF05 cells limits RVFV replication and growth. (**A**) RVFV rMP-12 RNA levels following stimulation of AF05 cells with commercial preparations of heat-killed *E. coli*, heat-killed *S. aureus*, or PBS (as control) were quantified by qRT-PCR. The bars represent the means of gene expression fold changes in the treated groups versus the controls +/− SEM, *n* = 3. Statistical analyses were performed on ΔΔCt values using unpaired t-tests. (**B**) RVFV titers in supernatants of PBS- or *E. coli*-treated AF05 cells were determined by plaque assay. Statistical analysis was performed using an unpaired t-test, *n* = 3.

Because we found Aag2-cell-derived AF05 cells to be more stress-resilient than our Aag2 cell isolate and because they do not change morphology upon bacteria treatment (not shown), the bacterial stimulation and viral infection assay were repeated for these cells. A decrease in viral RNA loads following treatment was still observed, which was statistically significant for the treatment using heat-inactivated *E. coli* (*p* = 0.02) (Figure 6A). In addition, pretreatment with *E. coli* also significantly limited RVFV titers, as determined by plaque assay (*p* = 0.017) (Figure 6B). We, thus, conclude that although the treatment of the Aag2 cells with bacteria also affected their cell morphology, the effects seen on RVFV replication and growth are likely due to immune signaling.

### 3.3. RVFV Infection Does Not Elicit an Immune Response in Infected Aag2 Cells

**Figure 7:**
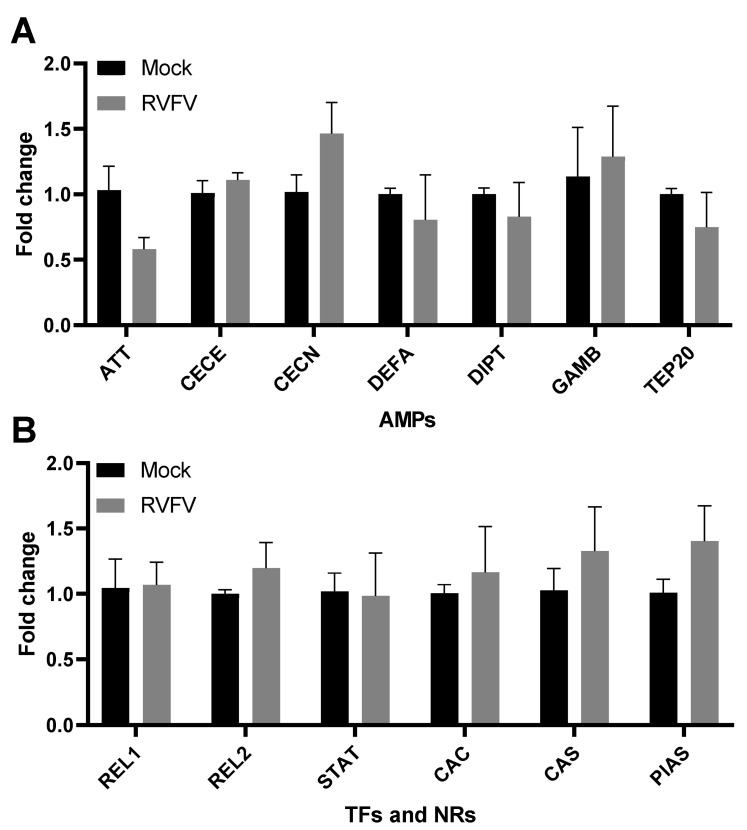
RVFV does not induce immune signaling in Aag2 cells. Aag2 cells were mock-infected or infected with RVFV rMP-12 at MOI 1 for 24 h, and the induction of genes encoding (**A**) AMPs or (**B**) transcription factors (TFs) and negative regulator (NRs) was quantified by qRT-PCR. The bars represent the mean gene expression fold changes in the RVFV-infected group versus the mock-infected group +/− SEM, *n* = 3. Statistical analyses were performed on ΔΔCt values using unpaired t-tests.

In order to investigate the effect of viral infection on *AMP* gene expression in Aag2 cells, the cells were infected with RVFV rMP-12. No significant changes to *AMP* gene expression were observed, indicating that RVFV does not directly induce innate immune signaling in this system (Figure 7A). The expression of *REL1*, *REL2*, and *STAT*, as well as of *CAC*, *CAS*, and *PIAS*, did not significantly change upon infection (Figure 7B). Because RVFV encodes NSm and NSs, which are proteins that are known to be functional (immuno) modulators, we considered the hypothesis that they might dampen the induction of immunity in Aag2 cells. However, infection of Aag2 cells with viruses lacking NSm or NSs equally did not induce the expression of *AMP*s (Figure S3), negating this hypothesis. 

We then repeated the infections with rBUNV, but again, we observed no significant impact of viral infection on immune signaling (Figure S4). Lastly, we determined that RVFV rMP-12 does not change the RNA levels of the insect-specific viruses CFAV and PCLPV, persistently infecting Aag2 cells (Figure S2C).

### 3.4. RVFV Infection of Aag2 Cells Primes Immune Responses to Bacteria

**Figure 8:**
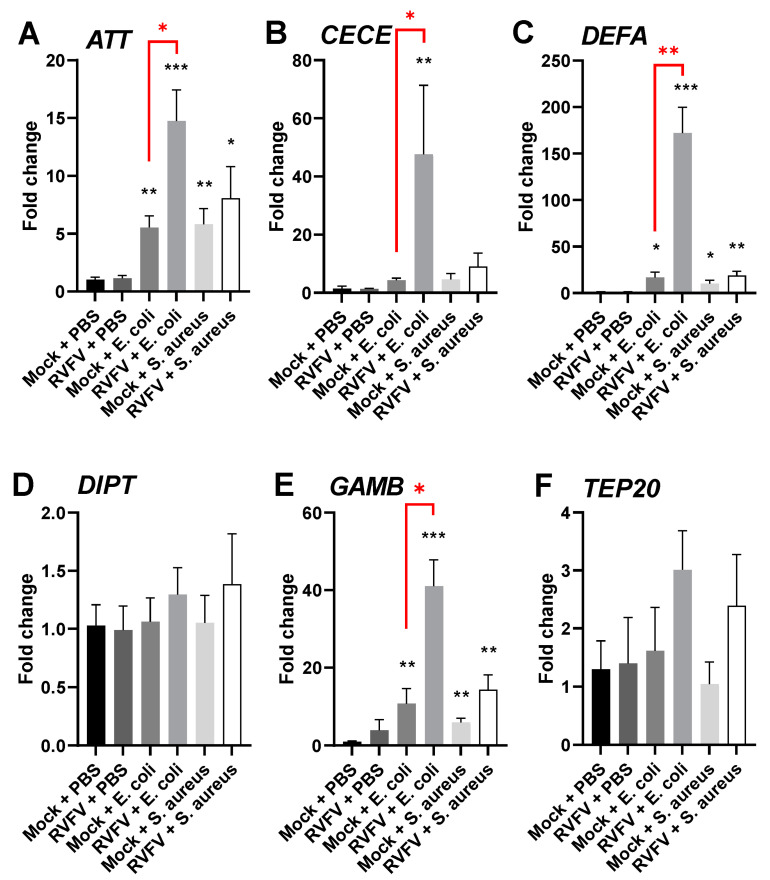
RVFV infection primes Aag2 cell immunity in response to bacteria. Aag2 cells were mock-infected or infected with RVFV rMP-12 at MOI 1 for 24 h, followed by treatment with PBS (control), heat-inactivated *E. coli*, or heat-inactivated *S. aureus* for 16 h, and the induction of *AMP*s was quantified by qRT-PCR. The bars represent the means of gene expression fold changes in the treated and/or infected groups versus the mock-infected, PBS-treated control group +/− SEM, *n* = 3. Statistical analyses were performed on ΔΔCt values either using Welch t-tests, assuming unequal variances when comparing the bacteria-treated groups to the mock-infected, PBS-treated control group, or unpaired t-tests when comparing RVFV-infected bacteria-treated groups to mock-infected bacteria-treated groups. Asterisks in black represent significant differences between the treated and/or infected groups and the mock-infected PBS-treated control group.

Next, we assessed the effect of RVFV infection on the responsiveness of the mosquito immune system to subsequent bacterial stimulation. Viral infection of Aag2 cells resulted in the enhancement of their immune responses to bacteria compared to uninfected stimulated controls (Figure 8). The enhancement was particularly prominent for *ATT* (*p* = 0.022), *CECE* (*p* = 0.012), *DEFA* (*p* = 0.003), and *GAMB* (*p* = 0.019) expression (Figure 8A–C,E), and was more pronounced for *E. coli* than for *S. aureus* treatment. In conclusion, the data suggest that RVFV rMP-12 infection is able to modulate the immune responses of Aag2 cells to external bacterial stimulation. Interestingly, no such enhancement was observed for rBUNV (Figure S4). 

### 3.5. RVFV Infection Regulates Expression of Selected Pattern Recognition Receptors

**Figure 9:**
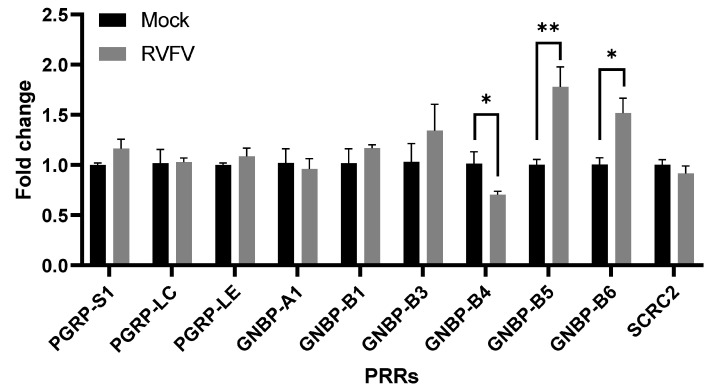
RVFV infection alters the gene expression levels of pattern recognition receptors in Aag2 cells. Aag2 cells were mock-infected or infected with RVFV rMP-12 at MOI 1 for 24 h, and the induction of the genes encoding pattern recognition receptors (PRR) was quantified by qRT-PCR. The bars represent the means of gene expression fold changes in the infected versus mock-infected cells +/− SEM, *n* = 3. Statistical analyses were performed on ΔΔCt values using unpaired t-tests.

In order to identify a molecular mechanism for the RVFV-dependent enhancement of antibacterial immune responses in Aag2 cells, we investigated whether RVFV rMP-12 may directly prime immunity by regulating the expression of PRRs involved in bacterial sensing, thus promoting immune recognition and signaling. We quantified the expression levels of the genes encoding the peptidoglycan recognition proteins (PGRPs)-S1, LC, and LE of Gram-negative binding proteins (GNBPs)-A1, A2, B1, B3–B6, and scavenger receptor C2 (full list of accession numbers available in Table 1), all of which are known to be involved in immune signaling and the production of AMPs. Although RVFV infection did not change the expression levels of the genes encoding PGRPs, we observed the significant downregulation of GNBP-B4 (*p* = 0.049) and the significant upregulation of GNBP-B5 (*p* = 0.009) and GNBP-B6 (*p* = 0.025) (Figure 9). GNBP-A2 had very low expression levels in our Aag2 cells, which did not increase upon RVFV infection (not shown) and has, thus, been excluded from the figure. Our data indicate that the modulation of PRR expression might be one mechanism by which RVFV infection primes immunity in Aag2 cells. 

## 4. Discussion

Its potential to emerge globally, together with the lack of effective treatment regimens, renders RVFV a pathogen of increasing public health concern. Studying the vector immune response to RVFV is essential for understanding the biology of the virus and the generation of transgenic mosquitoes with reduced transmission efficiency. 

The main vectors of RVFV are *Ae. vexans* and members of the *Culex pipiens* species complex. However, there is no available *Ae. vexans* cell line that could be used for in vitro studies, and the *Cx. quinquefasciatus* HSU cell line [59] does not propagate RVFV well (unpublished observation ID). *Ae. aegypti* mosquitoes are highly susceptible to RVFV infection in laboratory settings and support a high viral dissemination rate [60]. Hence, the Aag2 cell line derived from the embryos of *Ae. aegypti* mosquitoes was used in this study. It has previously been found to be a good model for immune studies [46,49]. We confirmed that immune signaling pathways and effector expression could be stimulated using both Gram-positive and Gram-negative bacteria. Given the inability of Aag2 cells to signal through the Toll pathway following bacterial stimulation alone ([9,46,53] and Figure S1), the observed signaling activity is likely to be mediated by the IMD and Jak-STAT pathways. Differences in the magnitude with which Aag2 cell isolates respond to immune challenges were observed (Figure 2), possibly due to differences in passage history and cell type composition within the cell isolates. 

Virus infection was sensitive to immune signaling, with lower RVFV rMP-12 and rBUNV RNA levels observed in bacteria-stimulated Aag2 and Aag2-AF05 cells, but not in cells treated with LPS, possibly due to the relatively low *AMP* induction levels following LPS treatment. The mechanisms by which immune pathway activation reduces RVFV and BUNV replication in Aag2 cells remain unclear. In mosquitoes, innate immune signaling and AMP production occur mostly in the fat body, with AMPs and other humoral factors being secreted into the hemolymph, but also locally in tissues, such as the midgut epithelium [8,61,62]. It is important to note that although *AMP* transcription is commonly used as a read-out for immune activation, AMPs are not necessarily directly antiviral. More importantly, immune signaling in infected tissues leads to the global activation of hemocyte-mediated humoral and cellular immunity, which ultimately enables the elimination of pathogens and infected cells. Because hemocyte cell populations are also present in the Aag2 cell line [49], they may get activated in response to bacterial challenge and confer an antiviral effect via the secretion of AMPs and other humoral factors, through the induction of RNAi or the phagocytosis of infected cells. Further, we observed modest activation of autophagy upon challenging the Aag2 cells with heat-inactivated *S. aureus* (Figure 4F). RVFV infection has been shown to activate autophagy in *D. melanogaster*, which limits viral replication [63]. Thus, one can speculate that autophagy contributes to a bacteria-induced reduction in RVFV RNA levels. 

We then demonstrated that the related bunyaviruses RVFV rMP-12 and rBUNV did not activate *Ae. aegypti* immune responses in our Aag2 cells. The RVFV MP-12 isolate is attenuated in mammalian cells and animals [41,64,65]. This attenuation is partly mediated by a mutation in the viral NSs protein [41,64], an otherwise potent inhibitor of the mammalian interferon response. However, MP-12 NSs retains major functions, such as the shut-off of host transcription, the inhibition of *IFN-β* gene induction, and the degradation of Protein kinase R (PKR) [66,67]. In order to exclude the possibility that the rMP-12 NSs protein lead to transcriptional shut-off or immune inhibition in our assays, which may explain the lack of *AMP* gene expression induction, we repeated the experiments using an NSs-deficient rMP-12 virus. This virus also failed to induce immune signaling (Figure S3), with the Ct values of individual *AMP* and *RPS7* housekeeping genes being essentially identical between all the experimental groups (not shown). Of note, NSs expression is readily lost during the passage of RVFV in Aag2 cells [68]. When taken together, these findings indicate that NSs may not be an important (immuno) modulator in Aag2 cells. The role of NSs in mosquitoes is less clear. One study recorded a modest decrease in the dissemination rate and day-14 titer of RVFV lacking NSs compared to wildtype virus in *Ae. aegypti*; however, this finding was not mirrored in *Cx. quinquefasciatus* mosquitoes [69]. Thus, more research is needed. Further, the RVFV NSm protein has been shown to have an essential role in promoting RVFV dissemination from the mosquito midgut [70,71], raising the possibility that NSm might be an antagonist of IMD signaling. However, no significant induction of *AMP* gene expression uponinfection of Aag2 cells with a virus lacking NSm was detected (Figure S3). rMP-12 NSm appears to be fully functional in mosquito cells and mosquitoes, as RVFV rMP-12 and the wildtype strain ZH-501 showed identical growth kinetics in Aag2 and *Cx. tarsalis* Ct cells, as well as similar infection, dissemination, and transmission rates in *Ae. aegypti* and *Cx. tarsalis* mosquitoes [72]. These findings justify the use of rMP-12 in this study. 

The RVFV rMP-12 infection of Aag2 cells resulted in the enhancement of immune responses to subsequent bacterial challenge when compared to the mock-infected stimulated controls. Contrarily, a similar study conducted using Semliki Forest virus (SFV; *Togaviridae*, Alphavirus) in *Ae. albopictus*-derived U4.4 cells showed that the virus suppresses the immunity of the infected cells to subsequent bacterial challenge [73]. This was also true during DENV infections of Aag2 cells [7]. Interestingly, the post-stimulation of RVFV infected cells with bacteria resulted in the pronounced upregulation of *ATT*, *CECE*, *DEFA,* and *GAMB* (Figure 8). This implies that RVFV modulates the immune response of *Ae. aegypti* cells to external bacterial stimulation. This may be an unintended consequence of the modulation of proviral or antiviral nonimmune pathways by RVFV, given that the virus is sensitive to immune signaling.

As a possible mechanism for the observed immune priming, we investigated whether RVFV infection altered the gene expression levels of pattern recognition receptors that are known to respond to pathogen challenge via Toll, IMD, or Jak-STAT signaling. For this analysis, we selected genes encoding peptidoglycan recognition proteins (*PGRP*)-*S1* (Toll and IMD pathways [47,74,75]), *PGRP-LC* and *-LE* (IMD pathway [26,27,32,75]), scavenger receptor C2 (*SCRC2*; Jak-STAT pathway and phagocytosis), and Gram-negative binding proteins (*GNBP*s)*-A1*, -*A2*, and *-B1*, *-B3-B6* (Toll pathway; see below). 

*SCRC2* is a Jak-STAT pathway response gene that is downregulated in *Ae. aegypti* upon pathway activation [14] and flavivirus infection [13] and is upregulated following the infection of *Ae. aegypti* with chikungunya virus (CHIKV; *Togaviridae*, Alphavirus) [76]. Interestingly, SCRC2 was shown to bind to the DENV envelope glycoprotein directly and to mediate *AMP* induction in the context of DENV infection, in particular *Defensins A*, *C,* and *D* and *Cecropins E* and *N* [77]. In flies and mosquitoes, among others, GNBPs form a family of several proteins that are able to bind to fungal β-1,3-glucan and bacterial LPS [78], Lysine-type PGN (present in cell walls of Gram-positive bacteria [79])**,** and DAP-type PGN [80]. Each GNBP has a defined antimicrobial specificity [81]. For example, in *An. gambiae*, GNBP-B4 senses *E. coli* and *S. aureus*, whereas GNBP-A2 binds to *E. coli* but is ineffective against *S. aureus* [81].

Indeed, following RVFV rMP-12 infection of Aag2 cells, we found a modest but significant downregulation of *GNBP-B4* and the upregulation of *GNBP-B5* and *GNBP-B6*, suggesting that RVFV could prime immunity to bacteria via the regulation of GNBP expression. *GNBP-B4* has been found to be expressed in the mosquito head, thorax, and Malpighian tubules. *GNBP-B5* is expressed exclusively in the thorax, and *GNBP-B6* is expressed in the head and thorax [62]. In arboviral challenges, CHIKV infection of *Ae. aegypti* midguts induced *GNBP-B4* [82], CHIKV and Zika viruses (ZIKV; *Flaviviridae*, Flavivirus) downregulated *GNBP-B6* in the salivary glands [48], and DENV upregulated *GNBP-B4* and *GNBP-B6* in postmidgut compartments (carcass) [8]. Of note, RVFV was found to upregulate *PGRP-S1* expression [83], an observation that we could not confirm under our experimental conditions. GNBPs signal mainly through the Toll pathway through interactions with PGRP-SA (PGRP-S1) [79]. However, they are also known to interact with other PGRPs, several of which can signal via the IMD pathway or trigger the prophenol oxidase cascade, leading to the production of AMPs and reactive oxygen species, respectively (reviewed in [84,85]).

The genetic modification of the immune pathways of vector mosquitoes is a powerful tool to limit the vector competence of mosquitoes to arboviruses. Transgenic mosquito lines with altered expression of single immune effectors have been shown to be viable and to affect arbovirus growth and the wider immune landscape [86,87,88]. RVFV differs from other arboviruses in that it is transmitted by many different mosquito species. Hence, the release of transgenic mosquitoes with a reduced RVFV transmission capacity would realistically need to be targeted in a species- and geography-dependent manner. Ideally, a common immune target would be identified for manipulation. When comparing available *Ae. aegypti* (Aag2 cell) [83] and *Cx. pipiens* transcriptomes in response to RVFV infection [89], there was little overlap in the response genes. This is because of differences between the mosquito species analyzed, differences in the antiviral responses of cells and mosquitoes, the virus strain and MOI used, and the post-infection time points chosen for any analysis. Thus, our ongoing work focuses on such matched comparisons of mosquito immune responses to RVFV. However, based on our findings, mosquitoes with enhanced IMD pathway responses may be suitable to suppress RVFV transmission more broadly.

## 5. Conclusions

Our findings demonstrate the complex interactions between vector mosquitoes, arboviruses, and microbiota and emphasize the need for further investigations. We have shown that the magnitude with which different isolates of Aag2 cells respond to immune stimulation varies, likely as a function of the differences in passage history and cell type differentiation. We observed that bunyaviruses, such as RVFV, were sensitive to immune signaling while not inducing the expression of *AMP* genes or altering the gene expression levels of Toll, IMD, or Jak-STAT pathway components. However, we found that the pre-infection of Aag2 cells with RVFV primed their immune stimulation. This immune priming may be due to changes in the expression of immune receptors. Our findings form the basis for producing transgenic mosquitoes with modulated immune systems and, hence, reduced capabilities for RVFV transmission to ruminants and humans.

## Figures and Tables

**Table 1:** qRT-PCR primers used in this study.

Gene Symbol *	Pathway	Vectorbase Gene ID	Forward	Reverse	Reference
Housekeeping Gene and Antimicrobial Peptides (*AMP*s)					
*RPS7*		AAEL009496	ccaggctatcctggagttg	gacgtgcttgccggagaac	[44]
*ATT*	Toll [45], IMD [46]	AAEL003389	aacaaaggaagaaatagcgccg	ccttttggccgctgaacag	This study
*CECE*	IMD [9,46]	AAEL000611	ctcgttctgctcatcgggtt	tccttccaatttcttccccagc	
*CECN*	Toll [46], IMD [46]	AAEL000621	tcttggttcttgtggccgtt	cttgccgaatttccacctgg	
*DEFA*	Toll [46], IMD [46]	AAEL003841	ctctgtgtaccgtggccatc	ctccggcagttcatcgaaaaga	
*DIPT*	IMD	AAEL004833	gaagtggaaccagcagtgtcc	tcgtcctgttgatgggtagct	
*GAMB*	Toll [46], IMD [46], Jak-STAT [46]	AAEL004522	agctgcctataccgatgctt	caataccgggctccatatgc	
*TEP20*	Toll [8], IMD [47], Jak-STAT [48,49], JNK [48]	AAEL001794	ggatcttgccgctactgatttg	cggtccaatcactgaaaagcc	
**Transcription factors** (**TFs**)					
*REL1*	Toll	AAEL007696/ AAEL006930	tctgcccaacaacctcatagt	tggtggcatttcttggtcga	This study
*REL2*	IMD	AAEL007624	acttatctcggccctctgga	tgatgttgcgtcgttcaatcg	
*STAT*	Jak-STAT	AAEL009692	ggcaacagttttccaatcgagg	Gactgggacgtttagcaatcg	
**Negative regulators** (**NRs**)					
*CAC*	Toll	AAEL000709	gaagtccaaggagcaacaacag	acggcaaggtgtaggtaagtat	This study
*CAS*	IMD	AAEL027860	gccagtgtgaagttttccagg	atgtccgacgcttccatcag	
*PIAS*	Jak-STAT	AAEL026694	acgacgagttctgcaatgact	tgtcgatggtggggatgga	
*PUC*	JNK	AAEL010411	cctggagtacaagcagatccc	gggcgtcctcaatgaattcga	
**Pattern recognition receptors** (**PRRs**)					
*PGRP-LC*	IMD	AAEL014640	cagttcgaagcagttaccgg	ccccgatgtgagcttgtaga	This study
*PGRP-LE*	IMD	AAEL027982	acgttaactccatcaccggt	gtccgccatttgacactatct	
*PGRP-S1*	Toll/IMD	AAEL009474	caagtggagcgacattggtt	aactcgatgccagccaattc	
*GNBP-A1*	Toll?/IMD [47]	AAEL007626	agtgaattatgtctcggcaca	cgaacagctttattccgggaa	
*GNBP-A2*	Toll?	AAEL000652	tggaaagatattgattcgcgc	cgcattagacccgaagcata	
*GNBP-B1*	Toll [47]	AAEL003889	gcaccctttacattcgtccc	ggtgcattgttcaacagggt	
*GNBP-B3*	Toll?	AAEL009176	accaacaaccgagcgaattc	agcaaccaatgtaggacgga	
*GNBP-B4*	Toll?	AAEL009178	agctgatgaaactggtgagga	ctttcacaaccatcccacgc	
*GNBP-B5*	Toll?	AAEL003894	accggtcaaattccttcgtg	tcgccaaattcatcaaccga	
*GNBP-B6*	Toll?	AAEL007064	tcaatctcaacgagggtccc	gtggatctgttctgcccaac	
*SCRC2*	Jak-STAT [14]	AAEL006361	tcccaagttccgatttgtgtca	aaatccgttatccacagccgat	
**Viruses**					
CFAV			tgatgcgtggtgattgacatg	tgcaagtagtctgtccggttc	This study
PCLPV N			tcccacgtcagatgcaaact	ttgtgttccttgggtgcctc	
CYV segment A			acctccaaaacaccgaacaag	cttccccataatgccacgttt	
RVFV N			tgccacgagtyagagcca	gtgggtccgagagtytgc	[5]
BUNV M			tcagacggtatagaaggggca	atcaaggagtgggaagccatc	This study

* Gene symbols and abbreviations used: *RPS7*—Ribosomal protein S7; *ATT*—Attacin; *CECE*—Cecropin E; *CECN*—Cecropin N; *DEFA*—Defensin A; *DIPT*—Diptericin; *GAMB*—Gambicin; *TEP20*—Thioesther-containing protein 20; *REL1*—Relish 1; *REL2*—Relish 2; *STAT*—Signal transducer and activator of transcription; *CAC*—Cactus; *CAS*—Caspar; *PIAS*—Protein inhibitor of activated STAT; *PUC*—Puckered; *PGRP*—Peptidoglycan recognition protein; *GNBP*—Gram-negative binding protein; *SCRC2*—Scavenger receptor C2; CFAV—cell fusing agent virus RNA; PCLPV—phasi charoen like phasivirus RNA; CYV—Culex Y virus RNA; RVFV—Rift Valley fever virus RNA; BUNV—Bunyamwera virus RNA.

## Data Availability

The data presented in this study are available in the article and supplementary materials.

## Supplementary Materials

The following supporting information can be downloaded at: www.mdpi.com/article/10.3390/pathogens12040563/s1, Figure S1: Immune signaling reporter assays in Aag2 cells show induction of IMD and Jak-STAT, but not Toll pathways in response to bacteria; Figure S2: RNA levels of the insect-specific viruses CFAV and PCLPV are not regulated in Aag2 cells or Aag2-AF05 cells upon bacterial challenge, or by RVFV infection; Figure S3: RVFV rMP-12 NSm and NSs proteins do not inhibit *AMP* gene expression following viral infection of Aag2 cells. Figure S4: BUNV does not induce immune signaling in Aag2 cells; Figure S5: BUNV infection does not prime Aag2 cell immunity in response to bacteria.
