# Supplementary material for: Rift Valley Fever Virus Primes Immune Responses in Aedes aegypti Cells
Source: Pathogens. 2023 Apr 6;12(4):563. doi: 10.3390/pathogens12040563 (PMC10146816; doi:10.3390/pathogens12040563)
Supplement: Supplementary file 1 [file pathogens-12-00563-s001.zip › pathogens-2265077-supplementary.pdf]

Laureti et al., 2023, Supplementary data.

Rift Valley fever virus primes immune responses in *Aedes aegypti* cells.

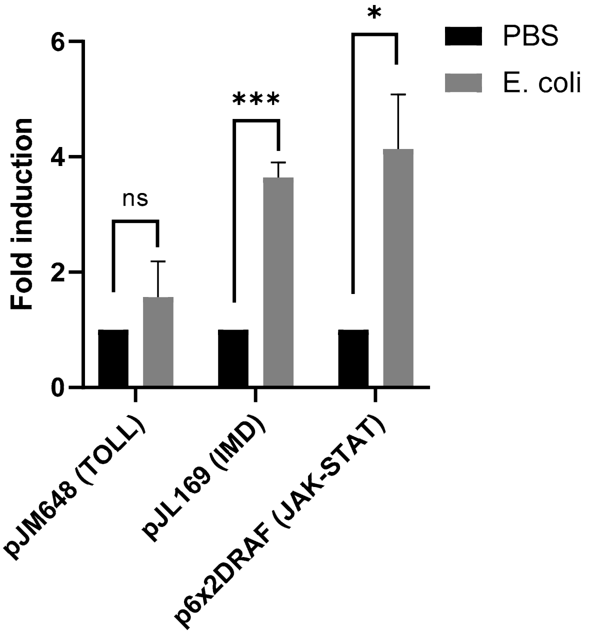

Figure S1. Immune signalling reporter assays in Aag2 cells show induction of IMD and JAK-STAT, but not Toll pathways in response to bacteria. Immune signalling reporter assays were performed by co-transfecting Aag2 cells with firefly luciferase (FLuc) expressing reporter plasmids pJM648 (Toll), pJL169 (IMD) or p6x2DRAF (JAK-STAT) and a *Renilla* luciferase (RLuc) plasmid as transfection normalization control. Cells were then treated with PBS as control or with heat-inactivated *E. coli*. Signalling activity was measured using a dual luciferase assay. FLuc measurements were normalized to RLuc measurements. Data is shown after subtraction of background luciferase levels and relative to PBS control. Bars represent means +/- SEM, *n* = 4. Statistical analyses were performed using Welch t-tests, assuming unequal variances.

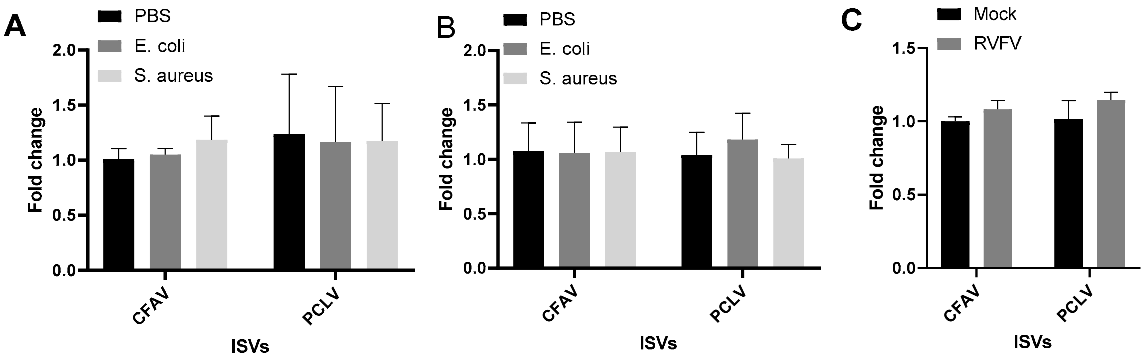

Figure S2. RNA levels of the insect-specific viruses CFAV and PCLPV are not regulated in Aag2 cells or Aag2-AF05 cells upon bacterial challenge, or by RVFV infection. **(A)** Aag2 cells were stimulated with heat-inactivated *E. coli* or *S. aureus* (prepared in-house), or were left unstimulated (PBS control) for 16 h, and RNA levels of CFAV and PCLPV were quantified by qRT-PCR. **(B)** AF05 cells were stimulated with commercial preparations of heat-killed *E. coli* or *S. aureus*, or were left unstimulated (PBS control) for 16 h, and RNA levels of CFAV and PCLPV were quantified by qRT-PCR. **(A)** and **(B)**: Bars represent mean gene expression fold changes in treated groups versus controls +/- SEM, *n* = 3. Statistical analyses were performed on ΔΔCt values using unpaired t-tests. **(C)** Aag2 cells were infected with RVFV rMP-12 at MOI 1 for 24 h, and RNA levels of CFAV and PCLPV were quantified by qRT-PCR. Bars represent mean expression fold changes in the RVFV-infected group versus the mock-infected group +/- SEM, *n* = 3. Statistical analyses were performed on ΔΔCt values using unpaired t-tests.

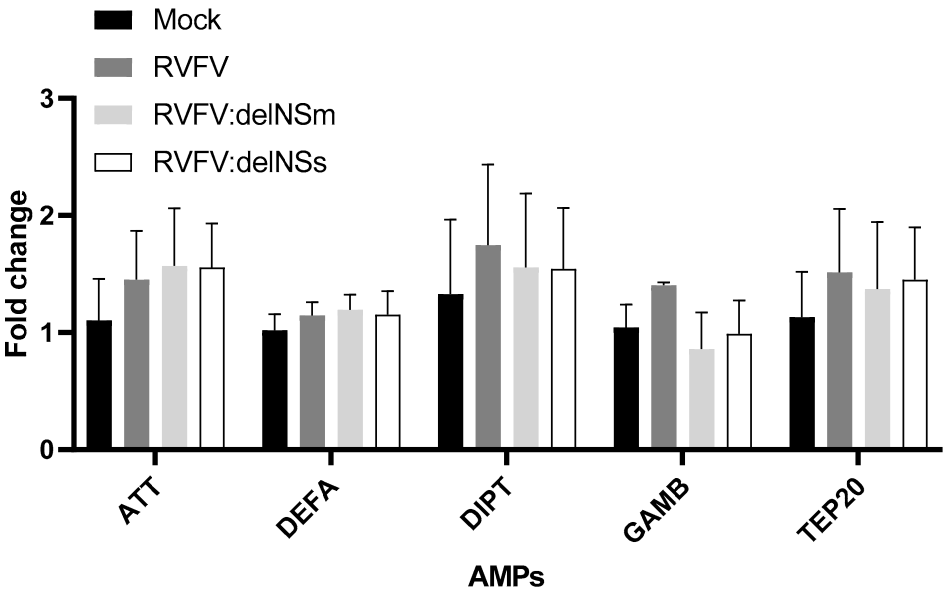

Figure S3. RVFV rMP-12 NSm and NSs proteins do not inhibit *AMP* gene expression following viral infection of Aag2 cells. Aag2 cells were mock-infected or infected with RVFV rMP-12, RVFV rMP12:delNSm or RVFV rMP-12:delNSs at MOI 1 for 24 h, and the induction of *AMP*s was quantified by qRT-PCR. Bars represent mean gene expression fold changes in the virus-infected groups versus the mock-infected group +/- SEM, n = 3. Statistical analyses were performed on ΔΔCt values using unpaired t-tests.

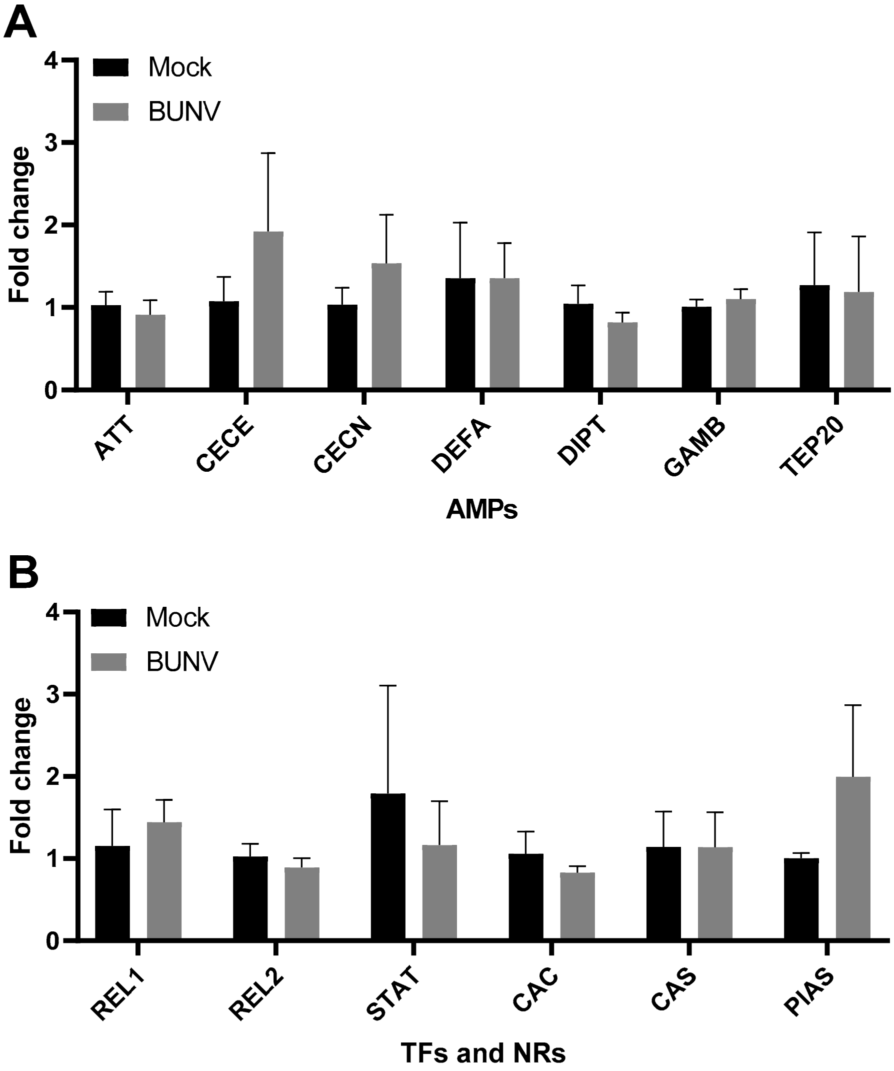

Figure S4. BUNV does not induce immune signalling in Aag2 cells. Aag2 cells were mock-infected or infected with rBUNV at MOI 1 for 24 h, and the induction of genes encoding **(A)** AMPs or **(B)** transcription factors (TFs) and negative regulators (NRs) was quantified by qRT-PCR. Bars represent mean gene expression fold changes in the BUNV-infected group versus the mock-infected group +/- SEM, $n = 3$. Statistical analyses were performed on ΔΔCt values using unpaired t-tests.

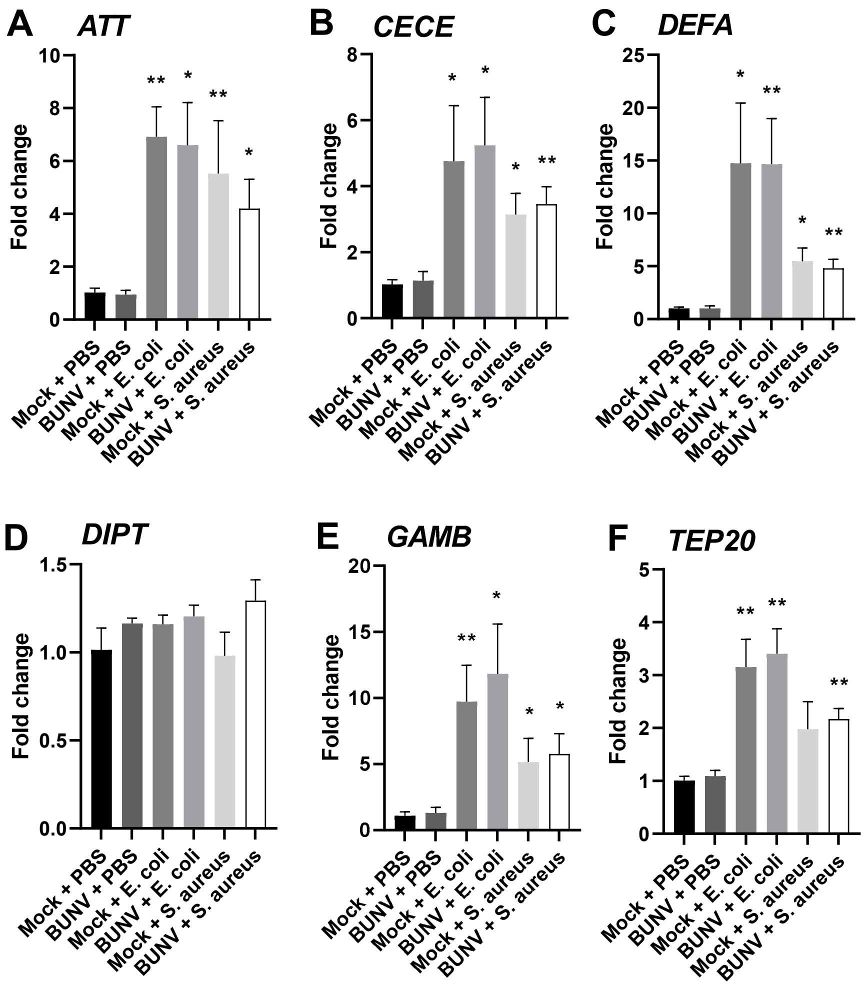

Figure S5. BUNV infection does not prime Aag2 cell immunity in response to bacteria. Aag2 cells were mock-infected or infected with rBUNV at MOI 1 for 24 h, followed by treatment of cells with heat-inactivated *E. coli*, heat-inactivated *S. aureus* or PBS as control for 16 h. The induction of *AMPs* was quantified by qRT-PCR. Bars represent means of gene expression fold changes in treated and/or infected groups versus the mock-infected, PBS-treated control group +/- SEM, n = 3. Statistical analyses were performed on ΔΔCt values either using Welch t-tests, assuming unequal variances when comparing bacteria-treated groups to the mock-infected, PBS-treated control group, or unpaired t-tests when comparing RVFV-infected, bacteria-treated groups to mock-infected, bacteria-treated groups. Asterisks represent significant differences between treated and/or infected groups and the mock-infected, PBS-treated control group.
